# Influence of distance to urban markets on smallholder dairy farming systems in Kenya

**DOI:** 10.1007/s11250-018-1575-x

**Published:** 2018-03-28

**Authors:** S. A. Migose, B. O. Bebe, I. J. M. de Boer, S. J. Oosting

**Affiliations:** 1 Animal Production Systems Group, Wageningen University & Research, PO Box 338, 6700 AH Wageningen, the Netherlands; 2 Department of Animal Sciences, Egerton University, PO Box 536, Egerton, 20115 Kenya

**Keywords:** Market quality, Production factors, Farm performance, Cattle functions

## Abstract

We studied influence of distance to urban markets on smallholder dairy farming system development. Farms were chosen from three locations that varied in distance to the urban market of Nakuru Town in the Kenyan highlands: urban location (UL, *n* = 10) at less than 15 km distance, mid-rural location (MRL, *n* = 11) in between 20 and 50 km west of Nakuru and extreme rural location (ERL, *n* = 9) beyond 50 km west and south-west of Nakuru. In-depth interviews with farmers and focus group discussions with eight groups of stakeholders were held to collect narratives and data about market quality, production factors, farm performance and functions of dairy cattle. We applied thematic content analysis to qualitative information by clustering narratives according to predefined themes and used ANOVA to analyse farm data. In UL, markets were functional, with predominantly informal market chains, with a high milk price (US $ 45.1/100 kg). Inputs were available in UL markets, but prices were high for inputs such as concentrates, fodder, replacement stock and hired labour. Moreover, availability of grazing land and the high opportunity costs for family labour were limiting dairy activities. In UL, milk production per cow (6.9 kg/cow/day) and per farm (20.1 kg/farm/day) were relatively low, and we concluded that farm development was constrained by scarcity of inputs and production factors. In rural locations (MRL and ERL), markets were functional with relatively low prices (average US $ 32.8/100 kg) for milk in both formal and informal market chains. Here, concentrates were relatively cheap but also of low quality. Fodder, replacement stock and labour were more available in rural locations than in UL. In rural locations, milk production per cow (average 7.2 kg/cow/day) and per farm (average 18.5 kg/farm/day) were low, and we concluded that farm development was constrained by low quality of concentrates and low price of milk. In all locations, production for subsistence was valued since income generated was used for non-dairy expenses. A tailor-made package of interventions that targets the above constraints is recommended for farm development.

## Introduction

Demand for milk is increasing globally because of rapid population growth, urbanisation and shifts in dietary patterns (Gerland et al. 2014). Milk demand in East Africa, for instance, is projected to increase by 43% in 2050 over the 2005/2007 base (Pica-Ciamarra et al. 2013). Because more than 70% of the future world population will live in cities by 2050, demand for milk will be concentrated largely around urban areas (Valin et al. 2014). This increased demand for milk in urban areas may influence dairy farming (Swain and Teufel 2017).

Our theoretical framework is that distance of farms to urban markets influences smallholders’ benefits from the increasing demand for milk. This is because distance to urban markets affects market quality for farm inputs and outputs (Chamberlin and Jayne 2013), and availability of production factors (Jiang et al. 2013), and therefore, influences levels of inputs and outputs (Staal et al. 2002). Market quality is defined as “the attractiveness and reliability of input procurement arrangements and output market chains” (adapted from Duncan et al. 2013) and is reflected in availability of inputs of good quality, reliability of suppliers and opportunities to sell products at favourable prices.

In urban location (UL), a high market quality is expected because of high demand and low transaction costs for outputs and inputs. Low transaction costs result, among others, from a well-developed and reliable infrastructure, such as roads and electricity supply (Chamberlin and Jayne 2013). Moreover, production factors, such as land and labour, are expected to be scarce and expensive because of high pressure on land for urban expansion and the availability of alternative well-paid labour opportunities (Jiang et al. 2013). Farms in UL, therefore, are expected to become intensive, i.e. to maximise profit per unit land or labour, with high levels of inputs and outputs. High input levels are reflected in use of improved exotic breeds, good-quality fodder (i.e. hay, maize silage or Napier (*Pennisetum purpureum*)) and concentrates and proper veterinary care (i.e. drugs, e.g. anthelmintic and acaricides, and treatment services). Because of high input and output levels, it is expected that farms will enter into formal value chains with specialised and efficient farming system designs and dairy cattle functions will be more oriented towards the production of milk at such high input-high output farms than at subsistence farms (Oosting et al. 2014).

In rural locations, in contrast, market quality is expected to be low, i.e. high input prices, because of low availability of inputs, unreliable supply and low output prices. This could be attributed to low demand and high transaction costs because of underdeveloped and unreliable infrastructure (Chamberlin and Jayne 2013, Gollin and Rogerson 2014). Moreover, production factors, land and labour, are relatively abundant and cheap because of lower population pressure and less job opportunities, respectively, than in urban areas (Jiang et al. 2013). Farms in rural locations, therefore, are expected to remain relatively extensive with low input-low output levels (Van Veenhuizen and Danso 2007). Because of low input-low output levels, farms are diversified and dairy cattle functions are manifold, such as production of manure, draught power, banking and insurance besides providing small daily income for household expenses (Oosting et al. 2014, Weiler et al. 2014). Specialisation under rural conditions involves risks and farms may remain subsistence to be resilient (ibid).

There is a knowledge gap on the influence of distance to urban markets on development of smallholder dairy farming systems. Duncan et al. (2013) studied effects of market quality on dairy intensification in India and Ethiopia and concluded that intensification was high when the market quality was high. This study of Duncan et al. (2013) classified market quality based on expert knowledge and functioning of formal and informal market chains, but did not analyse distance of farms to urban markets. The extent of importance of formal and informal market chains for milk marketing in urban and rural locations, besides, is unknown.

In the Kenyan highlands, smallholders produce milk in mixed crop-livestock systems (Van Leeuwen et al. 2012). The aim is subsistence and cattle in these systems have multiple functions: next to milk production, cattle also produce manure and traction and serve as a capital stock (Oosting et al. 2014, Weiler et al. 2014). Milk, exceeding household’s needs, is sold predominantly in informal market chains, directly to consumers or indirectly through retailers, such as vendors, brokers, middlemen and shopkeepers (Berem et al. 2015). Farms are distributed spatially and distances to urban markets are variable (Van de Steeg et al. 2010).

We use distance to urban markets as proxy for market quality with the objective to determine the influence of distance to urban markets on smallholder dairy farming system development. This is achieved through analysis of market quality, production factors, farm performance and dairy cattle functions. Results will inform designs of intervention packages to enhance benefits that the smallholder dairy farmers can obtain from the growing demand for milk in urban areas.

## Materials and methods

### Study area

Nakuru County was selected for this study because of four reasons. First, it is a major milk-shed, with high density of dairy cattle and smallholder dairy farmers (Van de Steeg et al. 2010). Second, it has an agro-ecological environment with bimodal rainfall and low temperatures, which is favourable for dairy production (Jaetzold and Schmidt 1982). Third, dairy production is a source of livelihood in (peri)-urban areas (Kashongwe et al. 2017b). Fourth, Nakuru Town, located within the county, provides an urban market for dairy products. Three locations were chosen that varied in distance to Nakuru Town: the urban location (UL) at less than 15 km distance, the mid-rural location (MRL) at a distance between 20 and 50 km west of town and the extreme rural location (ERL) at a distance beyond 50 km, west and south-west of town.

### Data collection

We conducted a cross-sectional survey to collect information at farm level. Subsequently, to complement the survey, we held focus group discussions (FGDs) with stakeholders to collect information at location level. To ensure a fair representation of smallholder dairy farms, we selected villages with a high proportion of smallholder dairy farms in UL (*n* = 10), MRL (*n* = 11) and ERL (*n* = 9). In each village, we selected one farm to represent the average smallholder dairy farm in the village (Creswell 2014). A small sample size was targeted, purposively, to suit the narrative methodology and to allow individual farmers to provide in-depth information about their perceptions on dairy farming. Each farm was visited once and in-depth interviews (IDIs), which lasted from 60 to 90 min, were conducted at the farmers’ homesteads (Creswell 2014).

For the FGDs, we established parallel groups, either consisting of farmers also involved in the survey and of non-farmer stakeholders. Each stakeholder group consisted of 7–10 members. In UL and ERL, we had one farmers’ group and one non-farmers’ group, whereas in MRL, we had two farmers’ and two non-farmers’ groups. The non-farmers’ group of stakeholders included representatives of government extension offices, agricultural research and training institutes, milk marketing groups, input suppliers, livestock non-governmental organisations and financial institutions. Each focus group met once and FGDs, which lasted for 6 h, were held at meeting venues located within the sub-county government offices.

A guide, with semi-structured questions, was developed and used to guide the process of data collection. The following topics were included in the guide: farm characteristics, such as farm size, land use and labour availability; management of dairy production, i.e. feeding, breeding and veterinary care; technical and economic performance; and dairy cattle functions. For the FGDs, participants were asked to discuss the topics and generalise responses for the whole location. FGDs were held to get stakeholder perception on dairy farming to corroborate, validate and put information from IDIs in a broader context. The IDIs and FGDs were conducted by the first author, assisted by livestock extension officers, who translated responses and facilitated selection of villages, farms and stakeholders. Audios and field notes were used to record interviews and discussions.

Data were collected between April and August 2013, under permit from the National Commission for Science, Technology and Innovation.

### Data analysis

The unit of analysis was the farm household for IDIs and the location for FGDs. Audio records of both IDIs and FGDs were transcribed and analysed qualitatively. Thematic content analysis (TCA) was applied to the narratives reflected in the transcripts (Boréus and Bergström 2017). The TCA had the following procedure: the narratives were read and elements of it were labelled by the first author using a predetermined thematic framework (Table 1). Each theme corresponded with questions in the interview guide, i.e. (i) market quality, (ii) production factors, (iii) farm performance and (iv) dairy cattle functions. Within each theme, we clustered responses according to issues that emerged. We made a summary of issues and used the summary to compare locations. Quantitative data collected during IDIs, furthermore, were used for assessment of input and output prices, production factors and farm performance. Numerical variables were converted to universal standard units: 88 Kenya shillings was 1 US dollar ($), 2.5 acres was 1 hectare (ha), 1 cattle of 250 kg was 1 tropical livestock unit (TLU) (Castellanos-Navarrete et al. 2015). Land size was calculated for land within location allocated to crops, fodder and non-farm activities and land outside the location. Communal grazing land was not included. The herd sizes comprised of cows (after first parturition, lactating and dry, av. 400 kg) and non-cows (heifers between 1 and 2 years, av. 200 kg, young stock between 4 and 12 months, av. 100 kg, and calves below 4 months, av. 50 kg). Milk yield was expressed in kg/cow/day, kg/herd/day, and kg/ha/day. Milk yield (kg/cow/day) was averaged by dividing total milk yield per farm (kg/herd/day) by the number of adult lactating cows and dry cows per herd. Milk consumed was included in the estimates of the yield per herd and dry cows were given zero yields. Milk yield (kg/ha/day) was calculated by dividing total milk yield per farm by total land size within the location per farm. Land outside the location was included only when it was used for fodder production. Communal grazing land could not be quantified. Dairy gross margins were calculated as dairy benefits (milk sold (kg/farm/day) × milk price (US $/kg) × 30 days) minus monthly production costs (feeds, hired labour, veterinary care and breeding). Milk consumed and opportunity cost for on-farm inputs (land, family labour, fodder, replacement heifers and bulls) were excluded. The aim was to get a cash flow. Variables were tested for normal distribution using QQ plots. A one-way ANOVA was used to test the location effect, followed by Fisher’s least significant difference (LSD) post hoc test to compare differences between means. Statistical analyses were done in the SAS software, v9.3©.

**Table 1:** Issues that emerged from thematic content analysis for market quality, production factors, farm performance and dairy cattle functions

Theme	Issues
Market quality	Functionality of milk and inputs markets
	Seasonality of price of inputs and milk
	Price of milk in informal and formal market chains
	Price and quality of inputs
Production factors	Land use for non-dairy activities
	Family labour use for non-dairy activities
Farm performance	Quality and quantity of inputs used
	Milk output level
	Economic performance
Function of dairy cattle	Importance of daily cash income for daily subsistence

## Results

Issues emerging from TCA were price and quality of inputs and outputs, scarcity of production factors, quality and quantity of input use, level of output and gross margin and subsistence functions of dairy cattle (Table 1).

### Urban location

#### Market quality

Individual farmers and FGD participants perceived milk and input markets as functional throughout the year. Morning and evening milk was sold, mainly via the informal market chains, direct to consumers or to shopkeepers and vendors. Milk prices were higher in the informal than formal market chain and in the lean season (i.e. season with the lowest milk production, which is towards the end of the dry season) than the flush season (i.e. the season with highest milk output in the beginning of the rainy season), when supply of milk from rural locations was in surplus. Only one farmer sold surplus milk through the formal market chain to a processor in the flush season. The assessment of output prices showed significantly higher prices of milk in UL than in rural locations (MRL and ERL) (Table 2).

**Table 2:** Price of milk and inputs, land size and herd size for farms in the in-depth interviews

Parameters	Urban location (*n* = 10)	Mid-rural location (*n* = 11)	Extreme rural location (*n* = 9)	RMSE
Price				
Milk (US $/100 kg)	45.1^a^	34.0^b^	31.7^b^	4.62
AI (US $/straw)	11.9 (*n* = 8)	11.0 (*n* = 10)	12.0 (*n* = 7)	1.75
Concentrates (US $/100 kg)	33.6^a^ (*n* = 6)	26.0^b^ (*n* = 6)	27.9^b^ (*n* = 8)	4.83
Land within location (ha/farm)				
Crops	0.4^a^	1.7^b^	1.7^b^	1.03
Fodder	0.4	1.4	1.2	1.47
Non-farm	0.2	0.5	0.5	0.54
Total	1.0^a^	3.6^b^	3.4^b^	2.14
Land outside location (ha/farm)^1^	5.3	–	–	7.67
Herd size (TLU)^2^				
Lactating cows	3.4	4.5	3.2	3.24
Dry cows	0.3	0.9	0.2	0.93
Heifers (1–2 years)	0.5	0.9	0.5	0.92
Young stock (< 1 year)	0.3	0.4	0.4	0.32
Total cattle	4.5	6.7	4.2	3.77

Values with different superscript are significantly different at *P* < 0.05

*RMSE* root-mean-square error, *AI* artificial insemination

^1^Land outside the location was allocated to fodder (0.8 ha) in only one farm

^2^1 tropical livestock unit (TLU) is 250 kg (Castellanos-Navarrete et al. 2015); hence, 1 cow was 1.6 TLU, heifer was 0.8 TLU and young stock was 0.3 TLU

Inputs, such as AI, veterinary care, concentrates, fodder, hired labour and replacement stock, were available. Prices were perceived as high for high-quality inputs, specifically high-quality concentrates and fodder in the dry season were expensive. The assessment of input prices revealed significantly higher prices of concentrate in UL than in rural locations (Table 2).

#### Production factors

Land and family labour were considered as scarce production factors since farmers preferred to allocate them to crops and off-farm activities. Only one farmer allocated more land to fodder than crops. Some farmers constructed rental housing on their land to get income. Total land size (owned or rented) and cropland size per farm available within the location were smaller in UL than in rural locations (Table 2). Some UL farmers owned or rented land in rural areas for crop production (on average 5.3 ha/farm) (Table 2). Family labour was available and was used for dairy activities but also for off-farm income-earning activities since six farms had at least one family member engaged in an off-farm job. Some farmers expressed that they hired labour for dairy activities while they had their family members working in an off-farm job. The herd size of cows and non-cows were considered as small, which was attributed to land and fodder scarcity. Herds were large (> 5 TLU, i.e. about three cows) in six farms. The herd sizes did not differ among locations (Table 2).

#### Farm performance

FGD participants mentioned that use of inputs, i.e. AI, high-quality concentrates and fodder, was high. In most farms, concentrates of high quality, veterinary care, fodder of low quality (crop residues and roadside grasses) and replacement stock from breeders were not used. Drug resistance and chronic cases of East Coast fever (ECF) were reported in some other farms, because of bad management. Moreover, use of expired drugs, unqualified veterinarians, conception failures and increased chances of bull calves due to late insemination were reported in some farms, which were attributed to poor delivery of services, such as veterinary care and AI. In some farms, however, high-quality inputs, such as hay, imported semen, replacement stock and hired labour, were used. Use of replacement stock of high genetic potential was discouraged by scarcity, high price and inability to achieve anticipated yields. Labour (family, hired or both) used was considered as of low quality because turn-over rate for hired semi-skilled labourers was high. Concentrates (see Table 3) and fodder were used in small quantities.

**Table 3:** Mean value for input use, milk production and economic performance of farms involved in the in-depth interviews

Performance	Urban location (*n* = 10)	Mid-rural location (*n* = 11)	Extreme rural location (*n* = 9)	RMSE
Input use				
AI (straw/cow/conception)	1.9	1.0	2.0	1.19
Concentrates (kg/cow/day)	1.7	1.2	1.4	1.11
Milk production				
Yield (kg/cow/day)	6.9	5.6	8.7	3.85
Yield (kg/herd/day)	20.1	20.1	16.8	17.96
Yield (kg/ha/day)^1^	68.6^a^	5.6^b^	8.7^b^	65.84
Economic performance				
Production cost (US $/month)	102.8	74.4	39.0	69.61
Dairy benefits (US $/month)	210.6	136.1	87.8	174.89
Dairy gross margins (US $/month)	107.8	61.8	48.8	127.41

Values with different superscript are significantly different at *P *<0.05

*RMSE* root-mean-square error, *AI* artificial insemination

^1^Total land per farm comprised of land for crops, fodder and non-farm land

Individual farmers and FGD participants perceived milk yield as low and attributed the relatively low milk production per cow to use of low-quality inputs in low quantities and to diseases, which they perceived to result in long lactation lengths and culling of cows when too old. Individual farmers and FGD participants perceived production costs as high because of high prices of inputs, such as feeds, water and hired labour, and high costs of breeding (repeated AI (see Table 3), flushing, hormone treatment) and veterinary care. Dairy benefits were perceived as low because of small herd sizes and low milk yield.

The farm assessment of performance, however, showed no differences among locations in input use, milk production and economic performance, except for milk yield per hectare, which was higher in UL than in rural locations (Table 3). Although average milk production was about 6.9 kg/cow/day, a production of 30 kg/cow/day at peak lactation was reported by one farmer. Production costs, for example, ranged from US $ 24 to 225 per month, dairy benefits from US $ 0 to 682 per month and dairy gross margins from US $ − 92 to 457 per month.

#### Dairy cattle functions

FGD participants mentioned that both commercial and subsistence functions were valued and dairy cattle generated daily cash income needed for daily household expenses. Only on some farms, the dairy income was used to purchase dairy inputs. Delivering milk to the formal market chain would give the farmer lower milk prices and would also mean that payment for milk would only be made weekly or monthly, which was not appreciated.

### Mid-rural location

#### Market quality

Individual farmers and FGD participants perceived milk and input markets as functional throughout the year, though road conditions during the rainy season sometimes limited access to markets. Morning and evening milk was sold, about equally distributed among formal and informal market chains at about same price. Some farmers could not sell their evening milk in the flush season, because the processor did not collect evening milk. Individual farmers and FGD participants perceived prices as low for milk in the flush and lean seasons, in both formal and informal market chains (see also Table 2, milk price in MRL lower than in UL). AI with semen produced within the country, concentrates, hired labour, fodder and replacement stock were perceived as available at low and acceptable prices. Hired labour for crop activities was scarce during cropping seasons. The assessment of input prices showed significantly lower concentrate prices in MRL than in UL (Table 2).

#### Production factors

Land and family labour were considered as scarce production factors, since farmers preferred to allocate more land to crops than to fodder. Seven farmers had grazing land but only two allocated more land to fodder than to crop farming. Some farmers rented land for crop production within MRL. Land size was larger in MRL than in UL (Table 2). Family labour was available but inadequate for dairy activities for some farmers. Family labour, besides, was spent more on crop than dairy activities, especially during cropping seasons, and cows that were not stall-fed or grazed on paddocks were tethered. Two farmers provided off-farm labour. The herd sizes were considered as small, which was attributed to scarcity of land for fodder. Herds were large in five farms.

#### Farm performance

FGD participants mentioned that use of concentrates and AI was high. Most farmers used small quantities of low-quality concentrates, inseminated cows with semen of local bulls and grazed cows. Moreover, replacement rates were low and replacement heifers were of zebu-exotic crossbreds. Veterinary care was infrequent and the quality of drugs and treatment services were low. Additionally, inadequate labour (of low quality because of lack of knowledge and old age), were reported by most farmers. Farmers attributed low levels of input use to scarcity, high price and insecurity and political unrest.

Individual farmers and FGD participants perceived milk yield per cow as low, which was blamed on use of inputs of low-quality and in low quantities. FGD participants perceived production costs as high because of high prices of concentrates and high costs of breeding (of repeated AI, three farmers used bulls to reduce AI costs). Dairy benefits were considered as low because of small herd sizes, low yields, low quantities of milk sold and low prices of milk. Dairy gross margins were perceived as low because of high production costs and low dairy benefits.

The assessment of farm performance, however, showed no differences among locations with regard to input use, milk production and economic performance, except for milk yield per hectare, which was lower in MRL than in UL (Table 3). Production costs ranged from US $ 10 to 306 per month, dairy benefits from US $ 0 to 511 per month and dairy gross margins from US $ − 22 to 216 per month.

#### Dairy cattle functions

FGD participants mentioned that the use of daily or weekly earning of a small amount of cash to supplement income from crops for household expenses was an important function of dairy cattle. In three farms, the dairy income was invested fully in dairy inputs. Additionally, delivering milk to the formal market chain not only gave low milk prices but also meant that payment for milk occurred monthly (instead of daily or weekly in the informal market chain), which was not appreciated. Generally, the formal market chain for milk was perceived as a more reliable chain than the informal one. Manure and cash income was used to support crop cultivation.

### Extreme rural location

#### Market quality

FGD participants perceived milk and input markets as functional throughout the year, though some farms did not sell their evening milk because the processor did not collect milk in the evening and because they preferred to consume the milk or to use it to rear calves. Market chains were mainly formal, and FGD participants perceived the milk price as low, specifically during the flush season. Most farmers purchased AI, concentrates and veterinary care, but fodder, replacement stock and labour were purchased only on some farms (e.g. four of the sampled farms) for two reasons. First, because own inputs were used and second, because fodder was scarce (e.g. hay for sale was scarce and Napier did not perform when temperatures were low), and experienced theft of replacement stock. FGD participants mentioned that prices of inputs were low for inputs of low quality. Access to markets was poor during rainy seasons because of bad roads and poor means of transport. The price assessment showed lower prices of milk and concentrates in ERL than in UL (Table 2).

#### Production factors

FGD participants mentioned that land and labour were scarce in ERL since they were allocated to cropping, but some farms allocated land to fodder production and some grazing occurred on communal lands. Only one farm allocated more land to fodder than crops. Non-farmer FGD participants, however, indicated that in some parts of the ERL (about 50%), land was available. Land size was larger in ERL than in UL (Table 2). Family labour was available for dairy activities, despite youth migrating to urban areas, use of labour for crop production and for employment as motorbike taxi driver. Only a few farms hired labour for dairy activities. The herds were considered as small, which was attributed to scarcity of grazing land. Herds were large only in two farms.

#### Farm performance

FGD participants mentioned that use of inputs was low, partly because of low availability and high prices. In most farms, low-quality concentrates (e.g. high bran content), veterinary care, grazing, unproven bulls and crossbred replacement heifers were used. The quantities of concentrates given were small and fodder was inadequate. The frequency of administration of anthelmintic drugs and spraying with acaricides were low. In most farms, cases of drug resistance, chronic conditions of ECF and death of animals were reported.

Individual farmers and FGD participants perceived milk yield as low and attributed the low milk production to use of inputs of low qualities and in small quantities, which contributed to diseases, prolonged lactations and culling of too old cows. FGD participants perceived production costs as high because of high prices of concentrates, and high cost of breeding (repeated AI), and of prevention and treatment of diseases. Dairy benefits were perceived as low because of small herd sizes, low yields, low quantities of milk sold and low prices of milk. Dairy gross margins were perceived as low because of high production costs and low dairy benefits. Production costs ranged from US $ 7 to 113 per month, dairy benefits from US $ 31 to 225 per month and dairy gross margins from US $ − 1 to 112 per month.

The assessment of farm performance showed no differences among locations with regard to input use, milk production and economic performance, but milk yield per hectare was lower in ERL than in UL (Table 3).

#### Dairy cattle functions

Individual farmers and FGD participants mentioned that the earning of a small amount of cash used to supplement crop income for household expenses was an important function of dairy cattle. Crop income was received after 4 months for potatoes and after 1 year for maize, while milk income from processors was received monthly. The dairy income was invested fully in dairy inputs on two farms only. Manure and cash income was used to support crop cultivation.

## Discussion

In Kenya, milk is produced predominantly by smallholder dairy farmers in the highlands, with a favourable climate for exotic breeds (Van Leeuwen et al. 2012). Milk, however, is sold to consumers mainly in major urban areas and in areas where sufficient milk production does not occur (Kenya 2010). Formal market chains are expanding because they provide an improved delivery of services and inputs at relatively low prices and are platforms for empowering farmers (Kilelu et al. 2013). Although, formal market chains are preferred in the national dairy master plan, the bulk (80%) of milk produced is sold through informal market chains. In this study, farmers were predominantly engaged in informal market chains, although we observed differences between locations. The informal market chains for milk were more important in UL than in rural locations because they offered high prices because of low transaction costs (explained in the introduction), i.e. low costs of transport and processing, mostly paid by buyers.

We determined the influence of distance to urban markets of smallholder dairy farming system development. Our theoretical framework was that distance to urban markets influences market quality and the availability of production factors, and which, therefore, influences levels of inputs and outputs. The low input-low output systems with quantities of inputs used and milk yields per cow being low in all locations, either close or far from urban markets was contrary. Signs of intensification, such as use of inputs of high-quality and high milk yield per hectare in response to land scarcity, however, were expected in UL.

Results for milk market were in line with our theoretical framework. Purchase of concentrates of higher quality at a higher price than those in rural locations may explain the price differences. Lack of differences in market quality between the rural locations is because shops that supplied inputs were present in local trading centres. In UL, however, market quality for inputs was negatively affected by lack of fodder, replacement stock and hired labour, also, reported in other studies (Gillah et al. 2012, Duguma et al. 2017). Variation in quality of inputs is a common observation in Kenya (Njoroge et al. 2015).

The observation of differences in production factors between UL and rural locations is in line with our framework and indicates that land and labour have high opportunity costs because of alternative uses (Jiang et al. 2013, Jayne et al. 2014). Lack of difference in production factors between rural locations, probably, is because the differences in distance between both rural locations to the urban market were not large enough to create variations in land holding and opportunity cost of labour.

Based on our observations that market quality and production factors differed among locations, we expected farming system development to differ between UL and rural locations such that UL farms become intensive high input-high output systems and farms in rural locations remain extensive. Results do not match our expectations. Low input use in UL is contrary to our expectations and to results from some studies in (peri)-urban areas in East African conditions (e.g. in Tanzania, Ethiopia, Kenya and Uganda) (Gillah et al. 2012, Duncan et al. 2013) but within range reported for peri-urban of Nakuru (Kashongwe et al. 2017b). A higher milk yield per hectare in UL than in rural locations reflects land scarcity and is in line with our framework (Jayne et al. 2014). Lack of differences in milk yield per cow among locations is contrary to our framework but agrees with results from Ethiopia and India (Duncan et al. 2013). Low yield in UL was unexpected and a milk yield of on average 6.9 kg/cow/day in UL (Table 3) is at the bottom end of the range of 6 to 20 kg/cow/day for dairy cattle in East African peri-urban smallholder farms (Gillah et al. 2012). Milk yield, however, varied widely (range 0 to 14.5 kg/cow/day) among farms. Dairy development policies often have the objective to develop relatively specialised, market-oriented high input-high output smallholder dairy farming systems. The UL farming systems in the present study are specialised and market oriented, but they have not become the high input-high output farming systems which we expected to develop (Oosting et al. 2014, Weiler et al. 2014). Possible reasons for this are several:

First is inadequate use of inputs, such as fodder, replacement stock and labour, because of scarcity in line with literature (Richards et al. 2016, Kashongwe et al. 2017b). On-farm production of inputs was limited by land and labour scarcity and trade of inputs was limited by high transaction costs (Gollin and Rogerson 2014). Other reasons for inadequate input availability may be related to low production for commercial purposes, seasonal availability and challenges related to conservation and storage of fodder. Fodder limitation was not mitigated by use of concentrates because in UL, as generally in Kenya, concentrates are used in minimal quantities because of perceived high prices and variability in nutritional content (Njagi et al. 2013). Crop residues and non-conventional feeds, usually used to supplement or substitute concentrates and fodder during feed scarcity, might be of low quality (Castellanos-Navarrete et al. 2015, Duguma et al. 2017). Poor husbandry and management might have occurred because of labour scarcity and inadequate capacity. Replacement stock used might have been of low genetic potential because smallholder farmers do not participate in genetic improvement programmes or due to unmet nutritional and management demand of high genetic potential (Ojango et al. 2016). High yields were observed for some individual cows but differences in daily yield among cows within farms, however, was not known because we calculated daily cow yield as herd yield divided by the total number of cows including dry cows with zero.

Second is diseases and reproductive problems, which prolong lactation lengths and calving intervals. Long calving intervals, further, contributed to few replacement stock and are associated with low replacement rates and culling at too old age (Baur et al. 2017). Farmers and stakeholders indeed reported a low milk yield due to long lactation lengths and milking of too old cows because of conception failures and associated low replacement rates. Veterinary care might have been inadequate for exotic cattle. Nevertheless, veterinary care is well adopted in Kenya (Kebebe et al. 2017).

Third is lack of opportunities to sell additional milk through informal market chains at high prices. Farms development to high input-high output systems, for example, moving from the current 20 to 40 kg/farm/day, would lead to saturation of informal market chains and force farmers to formal market chains (Duguma and Janssens 2014, Oosting et al. 2014).

Fourth is that the development of dairy into high output systems is at the expense of other cattle functions. In UL, cash income from daily sales of milk was valued for livelihood support. When faced with trade-offs, farmers are likely to prioritise livelihood over dairy investment. Current cash benefits are low and may be inadequate to satisfy both household needs and dairy investment. Additional or external sources of financing may be necessary to support family livelihood in order to save resources for purchase of inputs. Multi-functionality of dairy cattle is in line with literature (Weiler et al. 2014).

Other reasons reported by farmers and other studies might include old age of farmers which impairs physical and cognitive ability to adopt dairy improvement technologies; little knowledge which hinders use of correct inputs and husbandry management for improved breeds; and insecurity, such as theft and loss of property during political unrest, which interfere with management and use of replacement stock (Gillah et al. 2012).

Hence, in UL, high output is limited by scarcity of inputs, reproductive problems and saturation of informal market chains for milk and subsistence function of dairy cattle. Such multiple reasons imply that development into high output systems require a package of interventions including efficient input supply chains, formal market chains for milk with favourable prices and external financial sources (Oosting et al. 2014). Opportunities to improve fodder production and marketing include established fodder markets and formal market chains for milk as well as regulated standards for nutritional content of inputs in UL.

In rural locations, the observed level of milk yield was within expectations (Kashongwe et al. 2017a). The relatively low yield could possibly be attributed to use of zebu-crossbred cows of relatively low to medium genetic potential for milk. Grazing on communal land and crop fields after harvesting, besides, is providing only low-quality feeds (Kashongwe et al. 2017b). In rural locations, in addition, the low price of milk did not make it attractive for farmers to invest in dairy. Moreover, formal market chains were unable to collect evening milk, and dairy cattle was valued to support crop cultivation. Multifunctional benefits from dairy, such as manure and daily cash income, are important for farmers (Weiler et al. 2014). Specialisation, which often is a consequence of higher input use and higher production may cause a reduction of such multifunctional values and farmers may perceive this as negative (Oosting et al. 2014). Market quality for milk and inputs should be enhanced, either by establishing or strengthening producer organisations, to reduce transaction costs, secure milk delivery possibilities and increase empowerment of farmers (Kilelu et al. 2013).

## Conclusion

We related increased demand for milk in urban areas to smallholder dairy development at different distances from the urban market and found that, despite differences of milk prices and farm characteristics among locations, farms remained low input-low output and production for subsistence was valued in all locations. Farm development was constrained by scarcity of inputs and production factors in UL and low price of milk in rural locations. Dairy development interventions targeting high input-high output should address the key constraints.
